# Supplementary material for: Non-Small-Cell Lung Cancer Immunotherapy and Sleep Characteristics: The Crossroad for Optimal Survival
Source: Diseases. 2023 Feb 1;11(1):26. doi: 10.3390/diseases11010026 (PMC9944906; doi:10.3390/diseases11010026)
Supplement: Supplementary file 1 [file diseases-11-00026-s001.zip › diseases-2109297-supplementary.pdf]

diseases

MDPI

Communication

Non-Small-Cell Lung Cancer Immunotherapy and Sleep Characteristics: The Cross-road for Optimal Survival

Paul Zarogoulidis [1,2,*], Dimitrios Petridis [3], Christoforos Kosmidis [2], Konstantinos Sapalidis [2], Lila Nena [4], Dimitris Matthaios [5], Vasilis Papadopoulos [6], Eleni Isidora Perdikouri [7], Konstantinos Porpodis [8], Paschalis Kakavelas [9] and Paschalis Steiropoulos [10]

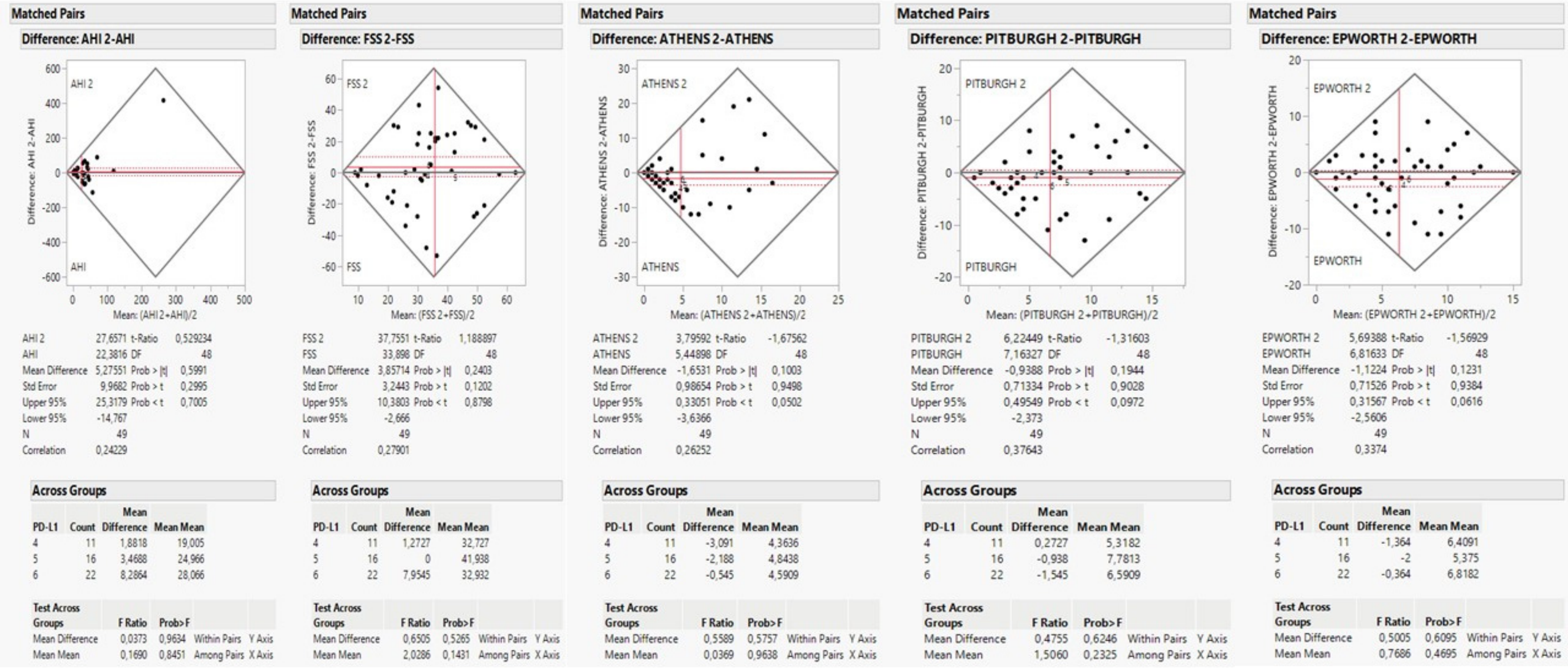

Figure S1. Turkey mean-difference plots, summary statistics and results of paired t-test of five questionnaire responses in accordance with PD-L1 test across groups. Horizontal and vertical lines determine the mean difference plus the 95% confidence intervals and the mean of paired sums accordingly.
